# Detection of Fine Radiographic Progression in Finger Joint Space Narrowing Beyond Human Eyes: Phantom Experiment and Clinical Study with Rheumatoid Arthritis Patients

**DOI:** 10.1038/s41598-019-44747-6

**Published:** 2019-06-12

**Authors:** Kazuki Kato, Nobutoshi Yasojima, Kenichi Tamura, Shota Ichikawa, Kenneth Sutherland, Masaru Kato, Jun Fukae, Kazuhide Tanimura, Yuki Tanaka, Taichi Okino, Yutong Lu, Tamotsu Kamishima

**Affiliations:** 1 Graduate School of Health Sciences, Hokkaido University, North 12 West 5, Kita-ku, Sapporo 060-0812 Japan; 2 Department of Radiology, NTT Sapporo Medical Center, South 1 West 15, Chuo-ku, Sapporo 060-0061 Japan; 3 Department of Mechanical Engineering, College of Engineering, Nihon University, Tokusada Aza Nakagawara 1, Tamura-cho, Koriyama 963-8642 Japan; 4 Department of Radiological Technology, Kurashiki Central Hospital, Miwa 1, Kurashiki, 710-8602 Japan; 5 Division of Photonic Bioimaging, Faculty of Medicine Research Center for Cooperative Projects, Hokkaido University, North 15 West 7, Kita-ku, Sapporo 060-8638 Japan; 6 Department of Rheumatology, Endocrinology and Nephrology, Faculty of Medicine and Graduate School of Medicine, Hokkaido University, North 15 West 7, Kita-ku, Sapporo 060-8638 Japan; 7 Department of Rheumatology, Hokkaido Medical Center for Rheumatic Diseases, Kotoni 1-3, Nishi-ku, Sapporo 063-0811 Japan; 8 Department of Radiological Technology, Sapporo City General Hospital, North 11 West 13, Chuo-ku, Sapporo 060-8604 Japan; 9 Faculty of Health Sciences, Hokkaido University, North-12 West-5, Kita-ku, Sapporo 060-0812 Japan

**Keywords:** Medical research, Rheumatoid arthritis

## Abstract

The visual assessment of joint space narrowing (JSN) on radiographs of rheumatoid arthritis (RA) patients such as the Genant-modified Sharp score (GSS) is widely accepted but limited by its subjectivity and insufficient sensitivity. We developed a software application which can assess JSN quantitatively using a temporal subtraction technique for radiographs, in which the chronological change in JSN between two radiographs was defined as the joint space difference index (JSDI). The aim of this study is to prove the superiority of the software in terms of detecting fine radiographic progression in finger JSN over human observers. A micrometer measurement apparatus that can adjust arbitrary joint space width (JSW) in a phantom joint was developed to define true JSW. We compared the smallest detectable changes in JSW between the JSDI and visual assessment using phantom images. In a clinical study, 222 finger joints without interval score change on GSS in 15 RA patients were examined. We compared the JSDI between joints with and without synovial vascularity (SV) on power Doppler ultrasonography during the follow-up period. True JSW difference was correlated with JSDI for JSW differences ranging from 0.10 to 1.00 mm at increments of 0.10 mm (R^2^ = 0.986 and P < 0.001). Rheumatologists were difficult to detect JSW difference of 0.30 mm or less. The JSDI of finger joints with SV was significantly higher than those without SV (P = 0.030). The software can detect fine differences in JSW that are visually unrecognizable.

## Introduction

Rheumatoid arthritis (RA) is a systemic inflammatory disease characterized by destructive synovitis^1,2^. Synovial inflammation promotes an immune response that causes articular cartilage degradation leading to joint space narrowing (JSN)^3,4^. “Tight control” and “treat to target” are treatment strategies for RA in which the aim is to achieve low disease activity or clinical remission with tailoring to the RA activity of an individual patient^5–7^.

For assessment of RA progression and therapeutic response, radiography is commonly utilized because it is superior to the other modalities in terms of simplicity, relatively low cost and high penetration rate^8–12^. Semi-quantitative ordinal scoring systems for the hand or foot such as the Sharp van der Heijde score (SHS) and the Genant-modified Sharp score (GSS) are currently the gold standard for assessment of radiographic progression in RA clinical studies^13,14^. However, these scoring methods have some disadvantages: they have insufficient sensitivity and suffer from inter- and intra-reader variations due to their subjective nature. They also require specialized training to use^15–18^. Over the past decade, because of early therapeutic intervention, radiographic RA progression has been remarkably reduced and it is necessary to detect subtle changes, especially in its early stages^19,20^, although the clinical relevance of such subtle radiographic changes which humans cannot recognize in each patient is not elucidated. Recently, computer-based methods to better evaluate JSN have been developed to overcome the disadvantages of traditional scoring methods^21–24^. Finckh *et al*.^25^ showed the correlation of JSN as defined by the conventional scoring method and the computer-based method. We developed an in-house software application that can detect joint space width difference between baseline and follow-up images, computing the joint space difference index (JSDI) by superimposing the two images^26^. In this software, extraction of distal contours in MCP joints is not required because the position of the metacarpal head relative to the phalanx was assessed when superimposing the images based on the phalanx. Previous studies have already demonstrated the high sensitivity and reliability of the software to detect JSN progression in RA patients^26–29^. However, the superiority over human observers of the software in terms of detecting fine JSW differences has not been clearly demonstrated.

To validate the JSW difference detectability of the software, both phantom and clinical images were used in the current study. We first developed a phantom joint made of titanium medical apatite (TMA)^30^ mimicking an MCP joint, directly connected to a micrometer serving as the gold standard of the JSW. The primary aim of this phantom study was to compare the smallest detectable changes in JSW between the JSDI and visual assessment. The second aim of this study was to test the software to detect fine differences in the joint space of RA patients with sustained clinical low disease activity (CLDA) which is not detectable with the GSS. This is based upon a clinical observation that JSN progression of RA patients is more prominent in joints with positive synovial vascularity (SV) than those without, assessed by power Doppler Ultrasonography (PDUS)^31–34^. To summarize, the purpose of this study is to prove that the software can detect fine radiographic progression in finger JSN more effectively than human assessment.

## Materials and Methods

### Phantom study

#### Phantom and Radiography

**Figure 1:**
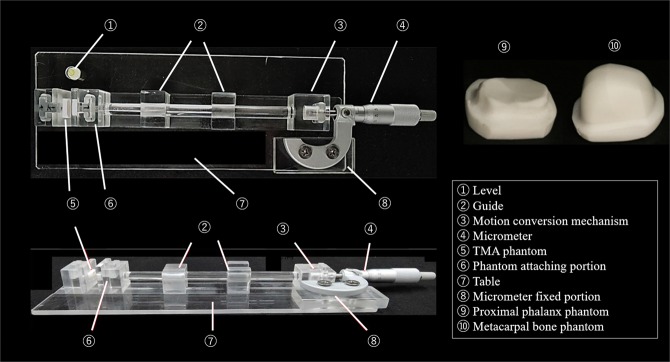
The TMA phantom which mimics the proximal phalanx and metacarpal bone, and the micrometer apparatus.

The phantom was made of TMA. TMA is a recently introduced material that is easier to model than hydroxyapatite, and its computed tomography (CT) value (Hounsfield unit) is nearly equal to that of cancellous bone^30^ (Table 1). The TMA was shaped into a metacarpal bone and a proximal phalanx. We developed a phantom joint which mimics an MCP joint, connected to a micrometer that can be adjusted arbitrarily and define the true JSW (Fig. 1). The JSW of the micrometer apparatus can be adjusted from 0 to 13.0 mm at increments of 0.01 mm. Pfeil *et al*.^35^ showed a close relationship between JSW and age and sex in healthy populations. In general, RA is more frequent in women and elderly people^2,36^. We therefore tested our ability to measure the true JSW from 1.20 to 2.20 mm at increments of 0.10 mm, and from 1.65 to 1.75 mm at increments of 0.01 mm, which were average JSW of second MCP joints in healthy elderly women^35^.

**Table 1:** Preparation of TMA material.

Proportion, TMA powder: Epoxy adhesive	1: 5
Sintering temperature	1370 K
Mean particle diameter	106 μm ≦
CT value	340 HU

CALNEO smart C47 (Fujifilm) was used to perform digital radiographs in this study. Acquisition parameters for the radiographs were identical to routine conditions: tube voltage = 50 kV; tube current = 100 mA; exposure time = 20 msec; source-detector distance = 100 cm. The X-ray beam was centered at the proximal edge of the phalanx phantom.

#### Image analysis

Manual assessment of the radiograph pairs was performed visually by two blinded rheumatologists, who have 10 and 15 years of experience in SHS or GSS, respectively. Radiograph pairs were created with JSW differences of 0.01 mm, and ranging from 0 to 0.40 mm at increments of 0.05 mm, and from 0.40 to 0.50 mm at increments of 0.02 mm between two radiographs. Ten pairs were prepared for each JSW difference. A pair of radiographs was selected at random and presented to the rheumatologists, whereupon they decided whether there was a difference in JSW between the radiographs. A total of 150 pairs of radiographs was judged for presence of JSW difference.

**Figure 2:**
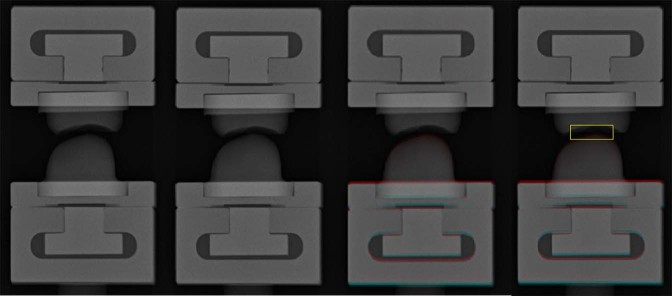
The radiographs of the phantom joint connected to micrometer apparatus. JSWs of the phantom joints were 2.2 mm (**a**) and 1.7 mm (**b**). In superimposed image (**c**), the JSW difference between two radiographs was detected. The rectangular ROI which was sized 60 × 20 pixels was located on the center of the joint space in the superimposed image.

We developed our own software, which can detect the JSW difference in two radiographs, with Microsoft Visual C++ 2015 using the Microsoft Foundation Class (MFC) Library (Microsoft). Two radiographs are read into the software, and the radiographs are then superimposed onto a single color image by assigning cyan to one image and red to the other image. If the pixel values in the two images are the same, the resulting pixel in the superimposed image is displayed as gray. When the margin of the proximal phalanx is superimposed accurately, the difference between the metacarpal head relative to the proximal phalanx between two images can be detected. The software visualizes JSW difference and quantizes it by computing the JSDI by setting a rectangular region of interest (ROI) centered on the joint space of the superimposed image. The JSDI was defined as the average absolute value of the difference of the pixel value in each pixel for two images inside the ROI (Fig. 2). The details of the software are presented in a previous article^26^.

The procedure for calculating JSDI was performed as follows: After two X-ray images were read by the software, a superimposed image was created. Each pixel value of one image was assigned cyan and each pixel value of the other image was assigned red automatically. Secondly, in phantom joints, the difference of the metacarpal head relative to the proximal phalanx was detected without extracting the contour of the proximal phalanx by superimposing the proximal phalanx of the images carefully. This operation was manual. Thirdly, a rectangular ROI with a size of 60 × 20 pixels was placed at the center of the phantom joint space in the superimposed image manually. Finally, the JSDI was automatically obtained by averaging the absolute value of the difference between each pixel value of the two images.

The JSDI was investigated twice using the software at the same JSW differences as in the visual assessment. Moreover, radiograph pairs were created with JSW differences ranging from 0.01 to 0.10 mm at increments of 0.01 mm, and from 0.10 to 1.00 mm at increments of 0.10 mm between the two radiographs. We investigated the JSDI between the pairs of radiographs using the software. These analyses were conducted by a radiological technologist.

### Clinical study

#### Patients

We recruited 15 patients who visited a local clinic for RA and were sustained long-term CLDA. All subjects satisfied the American Rheumatism Association 1987 revised criteria for the classification of RA. The patients had been treated with non-biologic disease-modifying antirheumatic drugs (DMARDs) (methotrexate [MTX], n = 8; MTX + tacrolimus, n = 3) or with biological DMARDs (MTX + adalimumab, n = 1; MTX + tocilizumab [TCZ], n = 2; TCZ monotherapy, n = 1). All patients underwent ultrasonography and radiography. The clinical characteristics of patients are shown in Table 2. The detailed patient population has been previously reported^34^.

**Table 2:** Clinical and laboratory characteristics of RA patients.

Characteristic	baseline	52nd week
Total no. of patients included	15	
Age, median (range) years	54 (32–69)	
Sex, female/male	13/2	
Duration of disease, median (range) months	50 (26–196)	
Duration of CLDA, median (range) months	15 (12–19)	
Swollen joint count, range	0–2	0–3
Tender joint count, range	0–2	0–3
DAS28-ESR, mean (SD)	2.03 (0.55)	1.96 (0.57)

*CLDA*; clinical low disease activity, *DAS28*; disease activity score with 28 joints, *ESR*; erythrocyte sedimentation rate, *SD*; standard deviation.

The study was conducted in compliance with the Declaration of Helsinki and received approval from the Ethics Review Committee at Faculty of Health Sciences, Hokkaido University. Informed consent was obtained from all patients.

#### Ultrasonography

All patients underwent PDUS of the first to fifth MCP and second to fifth PIP joints over the dorsal surface in the transverse at baseline and at the 8^th^, 20^th^ and 52^nd^ weeks. One of three US experts who specialized in musculoskeletal US (with experience in joint ultrasound for 12–18 years) and were blinded to other clinical information carried out the scans. A 13-MHz linear array transducer and US machine were used (EUP-L34P, Hl VISION Avius; Hitachi).

All PDUS images assessed SV by a quantitative PDUS method. A SV value was defined as the number of vascular flow pixels in the ROI. In previous studies, the quantitative PDUS method has already been shown to be valid and reliable to assess SV^31–34^.

#### Radiography

The radiographs of hands for RA patients were imaged at baseline and at the 52^nd^ week using Radnext 32 (Hitachi) under the following conventional conditions: tube voltage 50 = kV; tube current 100 = mA; exposure time = 25 msec; film focus distance = 100 cm. The X-ray beam was centered on the MCP joint of the second finger.

Each radiograph was examined for JSN progression by a rheumatologist experienced in GSS assessment for more than 15 years who was blinded to other clinical information according to GSS as follows: 0 = normal; 0.5 = subtle or equivocal narrowing; 1.0 = focal or mild narrowing; 1.5 = mild-to-moderate narrowing; 2.0 = moderate narrowing; 2.5 = moderate-to-severe narrowing; 3.0 = complete loss of joint space or dislocation in the presence of erosion; 3.5 = partial or equivocal ankyloses; 4.0 = definite ankyloses^37,38^. Joints were excluded if they were severely damaged (subluxation, ankylosed, and complete luxation). Scoring was repeated with more than 1 month’s interval to assess intra-reader agreement and reliability.

#### Software for JSN progression

**Figure 3:**
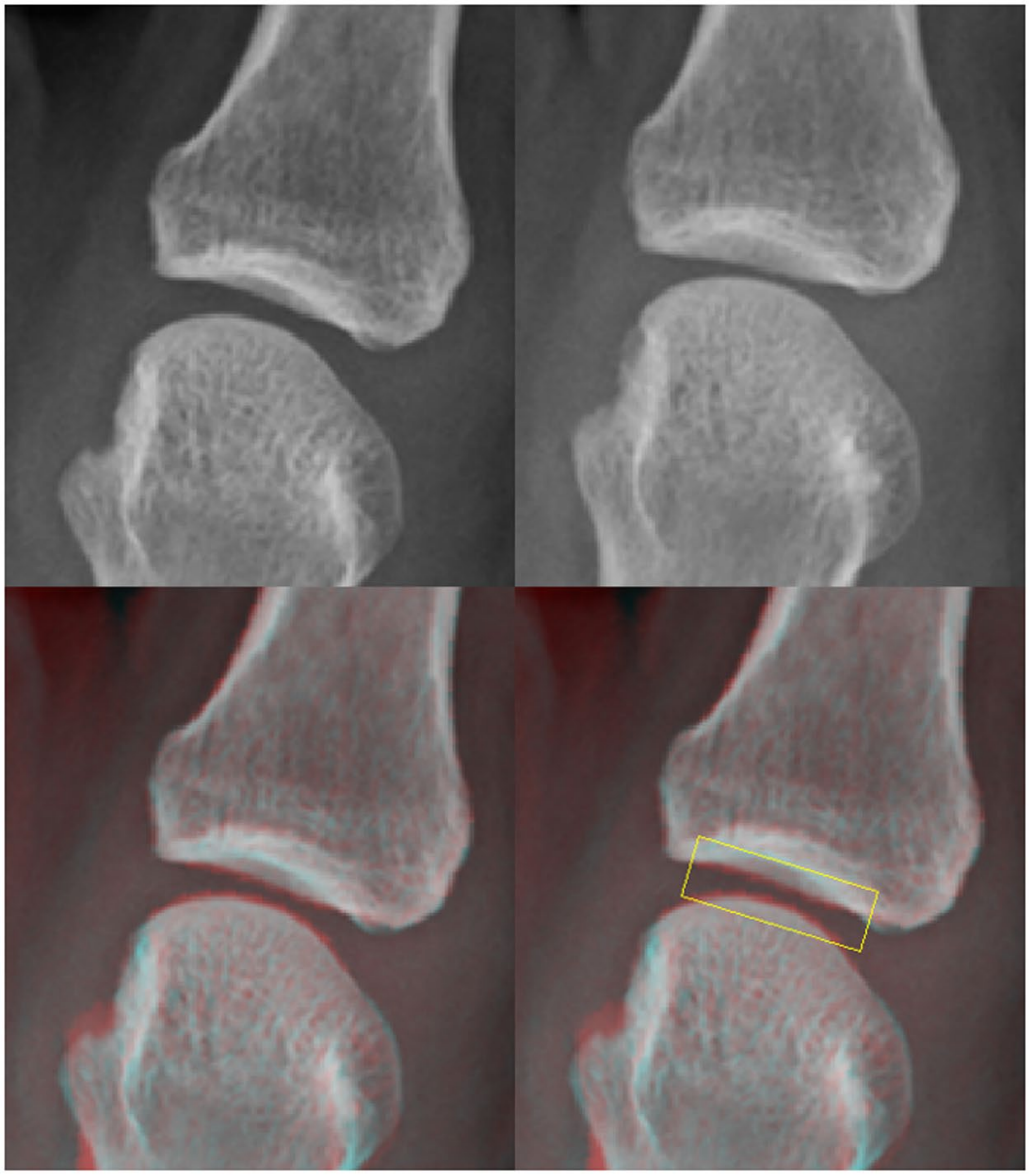
Clinical image analysis by the software. The radiographs of a 54-year-old female with RA. Second MCP joint for the left hand at baseline (**a**) and follow-up (**b**) are shown. In the superimposed image (**c**), the chronological change between the baseline and follow-up images was detected. The rectangular ROI which was sized 60 × 20 pixels was located on the center of the joint space in the superimposed image. The JSDI was calculated inside the ROI. The JSDI was 96.67 in this case.

The JSDI was obtained by averaging the absolute value of the difference between each pixel value of the baseline and follow-up images with the software (Fig. 3). Rectangular ROIs with sizes of 60 × 20 pixels and 25 × 7 pixels were used in MCP and PIP joints, respectively. Analysis was repeated with more than 6 months’ interval to assess intra-reader agreement and reliability by a radiological technologist.

### Statistical analysis

Statistical analyses were calculated with the use of IBM SPSS version 24.0 (IBM) for Windows (Microsoft). We calculated the correct assessment rate of JSW differences between radiograph pairs in visual assessment. The linear relationship between true JSW difference and JSDI was evaluated using a linear regression test. The smallest detectable difference (SDD) represents the smallest difference between two independently obtained measures which can be distinguished from measurement error. The SDD for JSDI was calculated according to the following formula:

$${\rm{SDD}}=1.96\times {{\rm{SD}}}_{{\rm{difference}}{\rm{JSDI}}}$$

where SD_difference JSDI_ was the standard deviation of the difference between the JSDI of two measurements^39,40^. The visual assessments of two rheumatologists were evaluated for inter-observer agreement using the kappa (κ) coefficient. The κ coefficient was interpreted as low agreement between 0 and 0.20, mild agreement between 0.21 and 0.40, moderate agreement between 0.41 and 0.60, good agreement between 0.61 and 0.80, very good agreement between 0.81 and 0.99, and perfect agreement at 1.00^41^. Intra-observer agreement of human and software analysis was estimated by calculating the intra-class correlation coefficients (ICC) employing a one-way random effect model for intra-observer agreement. ICC values were interpreted as poor agreement for values between 0 and 0.20, fair agreement for values between 0.21 and 0.40, moderate agreement for values between 0.41 and 0.60, substantial agreement for values between 0.61 and 0.80, and almost perfect for values between 0.81 and 1.00^42^.

We selected joints with a zero change of score on GSS (ΔGSS) from the finger joints in 15 RA patients. Two types of grouping were performed. First, we divided the joints into two groups: joints without SV during the observation period in terms of the PDUS (the SV [-] group) and those with SV at least once during the observation period in terms of the PDUS (the SV [+] group). We also categorized the joints into two groups: joints with therapeutic response (the response [R] group) and the remainder without therapeutic response (the no response [NR] group). We compared the JSDI between the SV (−) and SV (+) groups, and between the R and NR groups using the nonparametric Mann-Whitney *U* test. All tests were two-tailed and p values of <0.05 were considered to indicate a statistically significant difference.

### Compliance with ethical standards

The study was conducted in compliance with the Declaration of Helsinki and received approval from the local ethics committee. Informed consent was obtained from all patients.

### Guarantor

The scientific guarantor of this publication is Dr. Takeshi Saito.

### Statistics and biometry

No complex statistical methods were necessary for this paper.

### Informed consent

Written informed consent was obtained from all subjects (patients) in this study.

### Ethical approval

Institutional Review Board approval was obtained.

### Study subjects or cohorts overlap

Our patients’ population (15/15, 100%) has been previously reported [29, 33]. The purpose of the reported study [33] was to investigate the relationship between synovial vascularity and structural alternation assessed with conventional radiographic scoring of finger joints in rheumatoid patients with CLDA.

The other study [29] investigated the performance of a computer-based radiographic method by directly comparing with the conventional radiographic scoring method using the relationship between synovial vascularity and future structural alternation.

The current study further advanced the concept of the previous studies; to prove the superiority of the software in terms of detecting fine radiographic progression in finger JSN over human observers.

**Methodology**

- Secondary analysis of a prospective study
- Experimental (phantom)/observational (human)
- Performed at multiple institutions

## Results

Two rheumatologists judged a total of 150 pairs of radiographs for presence of JSW difference. The correct assessment rate of JSW differences between radiograph pairs is shown in Table 3.

**Table 3:** The correct assessment rate for each JSW difference between radiograph pairs.

JSW differences between radiograph pairs [mm]	Number of correct assessment		Percentage of correct assessment [%]
	Reader 1	Reader 2	
0.00	10	10	100
0.01	0	0	0
0.05	0	0	0
0.10	1	0	5
0.15	0	0	0
0.20	5	4	45
0.25	1	0	5
0.30	3	0	15
0.35	4	7	55
0.40	9	10	95
0.42	10	10	100
0.44	10	10	100
0.46	10	10	100
0.48	10	10	100
0.50	10	10	100

**Figure 4:**
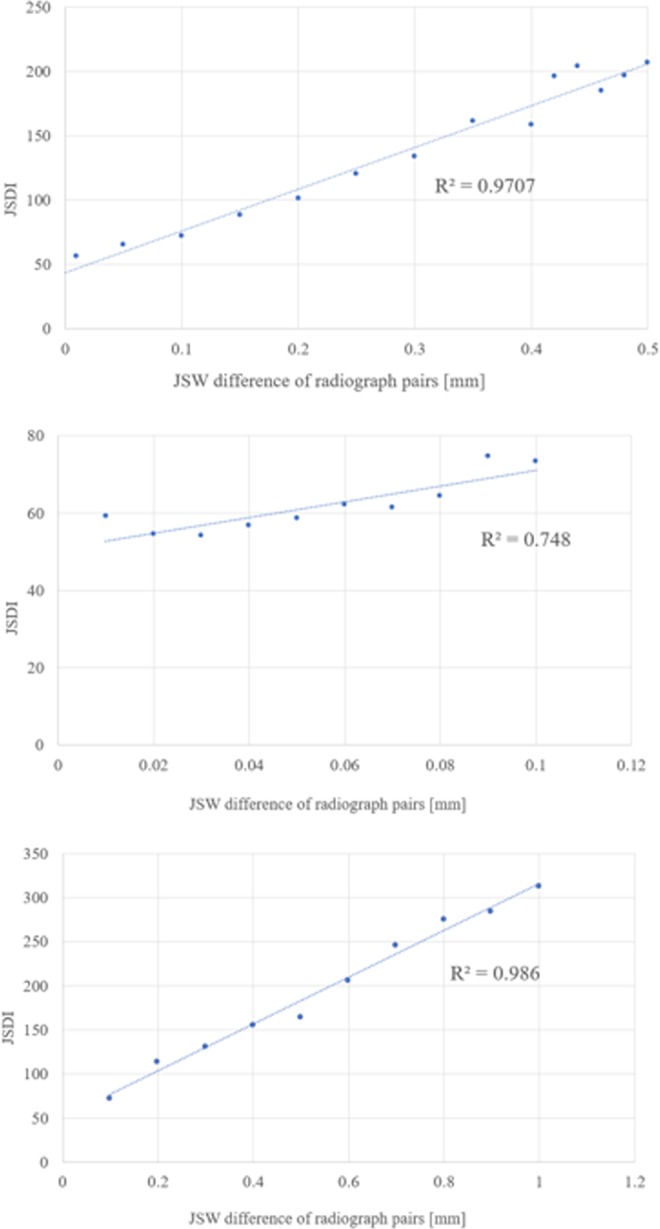
The linear regression between true JSW difference and JSDI for JSW differences ranging from 0.01 to 0.50 mm at the same JSW difference as visual assessment (**a**), ranging from 0.01 to 0.10 mm at increments of 0.01 mm (**b**), and ranging from 0.10 to 1.00 mm at increments of 0.10 mm (**c**).

The comparison between true JSW difference and JSDI is shown in Fig. 4. The linear regression test showed a significant correlation between true JSW difference and mean JSDI at the same JSW differences as visual assessment (R^2^ = 0.971 and P < 0.001). There were significant correlations between true JSW difference and JSDI for JSW differences ranging from 0.01 to 0.10 mm at increments of 0.01 mm (R^2^ = 0.748 and P < 0.01), and ranging from 0.10 to 1.00 mm at increments of 0.10 mm (R^2^ = 0.986 and P < 0.001). The calculated SDD for JSDI was 4.76. The software could correctly determine all JSW differences of 0.01 mm or more because all obtained the JSDI values greater than 4.76 of the SDD, which is a measure of the variation at a scale due to measurement error.

The κ coefficient of the inter-observer agreement for visual assessment was good agreement (κ = 0.786). The intra-observer reliability for JSDI was almost perfect (ICC = 0.999; 95% confidence interval [95% CI] = 0.999–1.000).

In the clinical study, out of 270 MCP and PIP joints in 15 RA patients, 259 finger joints were examined after excluding severely damage joints (subluxation, ankylosed, and complete luxation). Moreover, we targeted 222 joints with zero ΔGSS from 259 joints. We examined SV, GSS and JSDI of the 222 joints on PDUS and X-ray. The median age (range), median disease duration (range) and median CLDA duration (range) of RA patients were 54 (32–69) years, 50 (26–196) months and 15 (12–19) months, respectively. The median SV of the finger joints at baseline and at the 8^th^, 20^th^ and 52^nd^ weeks were the same (0 [interquartile range; IQR = 0–0]). Out of 222 joints, the number of SV (−)/SV (+) and R/NR joints were 191/31 and 202/20, respectively. The median GSS at baseline and at the 52^nd^ week were the same (1 [IQR = 1–2]). The mean ΔGSS (range) was 0 (0–0). The median JSDI was 53.4 (IQR = 38.8–74.0).

The JSDI of the SV (+) group was significantly higher than that of SV (−) group (P = 0.030). The JSDI of the NR group was significantly higher than that of R group (P = 0.002).

Intra-observer reliability for baseline GSS and follow-up GSS was in substantial agreement (ICC = 0.730; 95% CI = 0.668–0.782 and ICC = 0.718; 95% CI = 0.653–0.772, respectively). Intra-observer reliability for ΔGSS was in moderate agreement (ICC = 0.490; 95% CI = 0.392–0.577). Intra-observer reliability for JSDI was in almost perfect agreement (ICC = 0.963; 95% CI = 0.953–0.971).

## Discussion

In order to reveal the detectability of JSW difference in pairs of radiographs between the software and human observers, we first performed a phantom analysis. There was strong correlation between true JSW difference and JSDI for JSW differences between pairs of radiographs. We found that the software could detect JSW differences of 0.1 mm or less. In the phantom study, TMA was chosen because the CT value is nearly equal to that of cancellous bone. We also found that rheumatologists could detect JSW difference of 0.42 mm or more perfectly, while JSW difference of 0.30 mm or less were difficult to detect. The boundary where the JSW difference can be detected with human eyes was considered to be between 0.35 and 0.40 mm. Our results indicate that the software can detect finer JSW differences than human observers.

Clinical images of RA patients who underwent PDUS and X-ray were analyzed to prove that the software can also detect more subtle changes in JSN. Two hundred and twenty-two joints with no interval GSS change were examined and grouped based on the presence of SV because low symptomatic joints with imaging-proven synovitis are related to structural destruction^31–34^. We found that there was a significant difference between the JSDI of the finger joints with and without SV during the observation period. In addition, there was a significant difference between JSDI of the finger joints with and without therapeutic response. Fukae *et al*.^33,34^ reported that joint destruction occurred in joints with positive SV which occurred only once during the observation period. These results suggest that the software can detect fine differences in joint space which cannot be detected with the GSS.

Some computer-based methods to measure JSW from hand radiographs have shown to be useful because they were observer-independent and highly sensitive to change in JSN^22–24,43–48^. The software also had high sensitivity and reliability to detect JSN progression in RA patients^26–29^. However, these studies used the conventional method such as SHS or GSS, which is a visual assessment with ordinal score, as the gold standard. Although in a few “proof of concept” studies, performance of the software was directly compared with the conventional method^21,29^, this is the first study to prove the superiority of software over human observers, exclusively utilizing data which had no interval JSW difference determined by human observers. This highly sensitive method might shorten the period of clinical trials especially when the clinical relevance of such subtle radiographic changes which humans cannot recognize in each patient is established.

Huetink *et al*.^48^ studied the SDD of the JSW difference in a phantom joint with a width of 0.032 mm. In this study, the SDD of the JSDI was 4.76. This was somewhat lower than the JSDI value of 0.01 mm JSW difference between two radiographs. Therefore, we conclude that the JSN progression detectability of our software is higher than that in the study by Huetink *et al*.^48^. Hatano *et al*.^28^ reported that the inter- and intra-observer reliabilities using the software by non-experts were almost perfect (inter-observer ICC; 0.979 and intra-observer ICC; 0.976, respectively). The results of the reliability in the clinical section of this study were consisted with the previous study.

Several limitations to this study should be acknowledged. Foremost, the phantom joint was different from human finger joints. The TMA phantom was incompletely modeled on human finger bones, which are composed of cancellous bone and cortical bone. Furthermore, bones are surrounded by fluid and soft tissues rather than air. Second, the software was not designed to take into account the technical differences such as patient hand positioning and imaging conditions between radiographs. Although radiographs are made with standardized protocols, they may not be perfectly equal due to small differences in hand positions including difficulty in position/reposition of two 3D dimensional bones in a 2D image. The ‘human eye’ may correct for these changes, while the software cannot. We need to further manage variations in joint projection to improve measurement results on JSN progression; pixel value calibration between baseline and follow-up might improve the results. Third, only a small number of patients were included in this prospective clinical study. Further study is needed to confirm the results on a larger scale. Finally, the software relies partly on manual operations such as image registration of the baseline and follow-up images, and ROI location. We need to develop a fully automatic software application that can evaluate the JSN more easily and rapidly.

In conclusion, the software better detects fine radiographic progression in finger JSN than human eyes. Due to early diagnosis and treatment, radiographic RA progression encountered in daily practice can be subtle. Thus, fine JSN should be detected to evaluate therapeutic response. Our results indicate that as the software can detect radiographic progression more effectively than human observers, it might therefore be useful for assessment of RA at its early stages.
